# Diabetic Ketoacidosis With the Use of Alpelisib in a Patient With Metastatic Breast Cancer Without Diabetes

**DOI:** 10.1210/jcemcr/luae023

**Published:** 2024-03-22

**Authors:** Lakshmi Polisetty, Sneha Teresa Selvin, Jia Wei Tan

**Affiliations:** Bridgeport Hospital, Northeast Medical Group Internal Medicine, Bridgeport, CT 06610, USA; Saint Mary's Hospital, Waterbury, CT 06706, USA; Bridgeport Hospital, Northeast Medical Group Internal Medicine, Bridgeport, CT 06610, USA

**Keywords:** alpelisib, piqray, diabetic ketoacidosis, hyperglycemia, breast cancer, adverse drug events

## Abstract

Diabetic ketoacidosis (DKA) is a life-threatening medical condition. Alpelisib, a new drug used to treat phosphatidylinositol-4,5-bisphosphate 3-kinase catalytic subunit alpha mutated breast cancer, is reported to cause DKA as a rare adverse effect. We present a case of alpelisib-induced DKA in a patient with metastatic breast cancer without diabetes. An 81-year-old female with a history of hormone receptor-positive, human epidermal growth factor receptor 2-negative metastatic breast cancer presented to the emergency room with clinical features and blood work consistent with DKA. She was started on alpelisib 6 weeks before her presentation to the hospital. She did not have a documented history of diabetes. Upon admission, alpelisib was held, and her blood glucose returned to baseline with intravenous insulin and hydration. Post-discharge, she was managed with sitagliptin. Subsequent attempts to reintroduce alpelisib were associated with hyperglycemia, which led to the permanent discontinuation of alpelisib and the transition to alternative treatment options. Alpelisib causes hyperglycemia by inhibiting the phosphatidylinositol 3-kinase/activated protein kinase-B pathway, which regulates blood glucose levels. This case report illustrates DKA as a presenting symptom and provides potential management options for alpelisib-induced DKA. Hyperglycemia is a frequent adverse effect of alpelisib in patients with diabetes. This case report is unique as our patient developed uncontrolled diabetes within a few weeks after initiation of alpelisib.

## Introduction

Breast cancer is the most frequently diagnosed cancer globally. It is the leading cause of cancer-related death among women in the United States, second only to lung cancer (1). The most common form of breast cancer is hormone receptor-positive and human epidermal growth factor receptor 2 (HER2)-negative type (over 70%) (2, 3). In 2019, the Food and Drug Administration approved using alpelisib, a specific inhibitor of phosphatidylinositol 3-kinase (PI3K) alpha subunit, along with fulvestrant for managing hormone receptor-positive, HER2-negative, PIK3CA-mutated breast cancer in postmenopausal women and men (3). Despite significant improvement in progression-free survival, alpelisib treatment is associated with adverse effects, the most common being hyperglycemia. Diabetic ketoacidosis (DKA) is noted to be a rare adverse effect of alpelisib (2). DKA is a medical emergency characterized by hyperglycemia, electrolyte derangements, metabolic acidosis, and ketonemia (4). We present a case report of DKA as a presenting symptom in a patient without diabetes while on alpelisib therapy. The mechanism of alpelisib-induced hyperglycemia and the management protocol are also discussed.

## Case Presentation

An 81-year-old female with no prior history of diabetes mellitus presented to the emergency department with worsening fatigue and urinary frequency for 2 days. She endorsed polydipsia and polyuria for 2 weeks. She denied any fever, chills, cough, dyspnea, or gastrointestinal complaints.

## Diagnostic Assessment

Her medical history was only significant for metastatic estrogen receptor-positive, progesterone receptor-negative, and HER2-negative breast cancer with bone involvement. Breast cancer was diagnosed in 1989, and she underwent a left mastectomy and adjuvant chemotherapy. In 2017, she was found to have metastasis to bone. Despite receiving multiple lines of hormonal therapy and chemotherapy, her disease progressed, and in early December 2021, she was started on alpelisib and exemestane. A low dose of alpelisib was initiated to ensure tolerability and was up titrated from 150 to 300 mg over 3 weeks.

**Figure 1.**
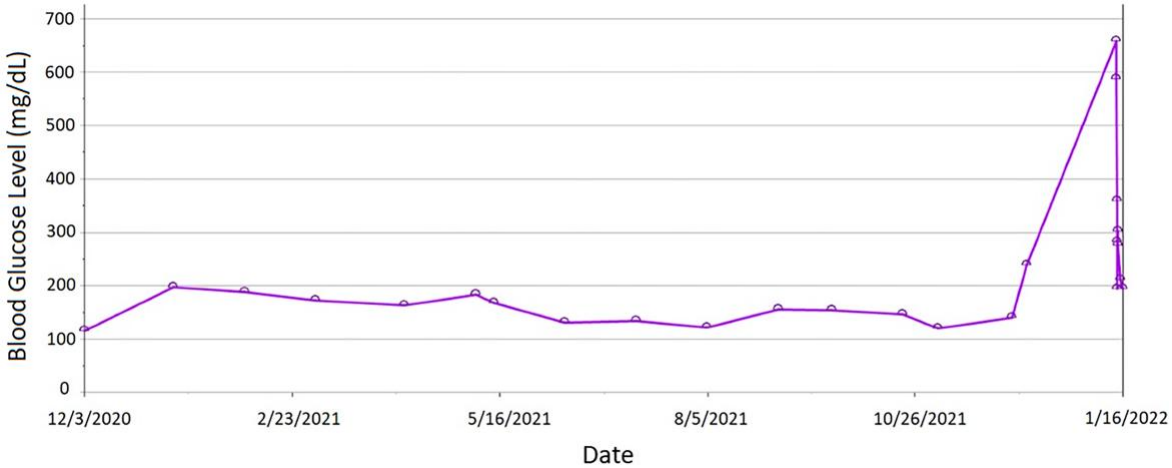
Graphic representation of blood glucose level trend of the patient at various outpatient and inpatient visits over the past 2 years.

Her glycosylated hemoglobin (HbA1c) was 5.5% 2 weeks before the initiation of alpelisib. Six weeks after starting alpelisib, she presented with worsening fatigue, polydipsia, and polyuria to the emergency room. Her labs revealed high blood glucose [659 mg/dL (36.5 mmol/L)] with anion gap metabolic acidosis [anion gap 21 mmol/L (21 mEq/L)], venous blood gas pH 7.26, 1+ ketonuria, and 4+ glucosuria. Figure 1 displays the blood glucose trends of the patient at various outpatient and inpatient visits. Blood work at admission and post-discharge follow-up is shown in Table 1.

**Table 1.** Blood report of the patient on the day of admission, on the day of discharge, and 4 days post-discharge

Lab	On admission	On the day of discharge	4 days post-discharge	Reference range
Blood glucose	659 (mg/dL) (36.5 mmol/L)	195 (mg/dL) (10.8 mmol/L)	213 (mg/dL) (11.8 mmol/L)	70–100 (mg/dL) (3.8–5.5 mmol/L)
Anion gap	21 (mmol/L) (21 mEq/L)	9 (mmol/L) (9 mEq/L)	11 (mmol/L) (11 mEq/L)	7–17 (mmol/L) (7–17 mEq/L)
C-peptide	4.3 (ng/mL) (1.4 nmol/L)			1.1–4.4 (ng/mL) (0.36–1.4 nmol/L)
Sodium	129 (mmol/L) (129 mEq/L)	139 (mmol/L) (139 mEq/L)	138 (mmol/L) (138 mEq/L)	136–144 (mmol/L) (136–144 mEq/L)
Potassium	4.6 (mmol/L) (4.6 mEq/L)	3.7 (mmol/L) (3.7 mEq/L)	3.9 (mmol/L) (3.9 mEq/L)	3.3–5.5 (mmol/L) (3.3–5.5 mEq/L)
Chloride	91 (mmol/L) (91 mEq/L)	110 (mmol/L) (110 mEq/L)	108 (mmol/L) (108 mEq/L)	98–107 (mmol/L) (98–107 mEq/L)
CO_2_	17 (mmol/L) (17 mEq/L)	20 (mmol/L) (20 mEq/L)	19 (mmol/L) (19 mEq/L)	20–30 (mmol/L) (20–30 mEq/L)
BUN	60 (mg/dL) (21.4 mmol/L)	18 (mg/dL) (6.4 mmol/L)	18 (mg/dL) (6.4 mmol/L)	8–23 (mg/dL) (2.8–8.2 mmol/L)
Creatinine	2.83 (mg/dL) (250 micromol/L)	1.50 (mg/dL) (132.6 micromol/L)	1.48 (mg/dL) (130.8 micromol/L)	0.4–1.30 (mg/dL) (35.3–114.9 micromol/L)
BUN/creatinine ratio	21.2	12	12.2	8.0-23.0
Calcium	9.6 (mg/dL) (2.4 mmol/L)	7.9 (mg/dL) (1.9 mmol/L)	9.2 (mg/dL) (2.3 mmol/L)	8.8–10.2 (mg/dL) (2.2–2.55 mmol/L)
Total bilirubin	0.6 (mg/dL) (10.26 micromol/L)		0.9 (mg/dL) (15.39 micromol/L)	≤1.2 (mg/dL) (≤20.52 micromol/L)
Alkaline phosphate	70 (U/L) (70 U/L)		54 (U/L) (54 U/L)	9–122 (U/L) (9–22 U/L)
Alanine aminotransferase	17 (U/L) (17 U/L)		18 (U/L) (18 U/L)	10–35 (U/L) (10–35 U/L)
Aspartate aminotransferase	21 (U/L) (21 U/L)		29 (U/L) (29 U/L)	10–35 (U/L) (10–35 U/L)
AST/ALT ratio	1.2		1.6	
Total protein	7.5 (g/dL) (75 g/L)		6.3 (g/dL) (63 g/L)	6.6–8.7 (g/dL) (66–87 g/L)
Albumin	4.4 (g/dL) (44 g/L)		3.7 (g/dL) (3.7 g/L)	3.6–4.9 (g/dL) (36–49 g/L)
Globulin	3.1 (g/dL) (31 g/L)		2.6 (g/dL) (26 g/L)	2.3–3.5 (g/dL) (23–35 g/L)
A/G ratio	1.4		1.4	1.0–2.2
b-Hydroxybutyrate	3.82 (mmol/L) (39.77 mg/dL)			0.27 (mmol/L) (2.81 mg/dL)
Glutamic acid decarboxylase	<5 (IU/mL) (<0.02 nmol/L)			<5 (IU/mL) (<0.02 nmol/L)
IA-2 antibody	<5.4 (U/ml) (<0.02 nmol/L)			<5.4 (U/ml) (<0.02 nmol/L)
Insulin autoantibody	<0.4 (U/ml) (<0.4 U/ml)			<0.4 (U/ml) (<0.4 U/ml)

Abbreviations: A/G, albumin/globulin; ALT, alanine transaminase; AST, aspartate transaminase; BUN, blood urea nitrogen; CO_2_, carbon dioxide; HbA1c, glycosylated hemoglobin; IA-2 antibody, islet antigen-2 antibody.

## Treatment

**Figure 2.**
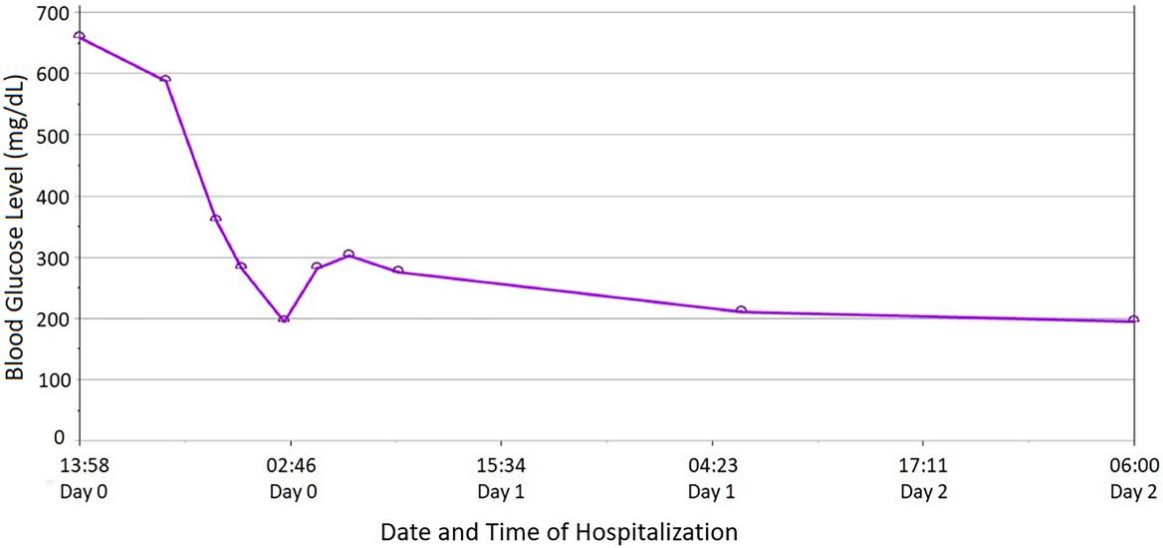
Graph showing rapid correction of blood glucose after prompt discontinuation of alpelisib.

She was admitted to the step-down unit for DKA management (intravenous hydration and insulin drip at 0.1 U/kg/hour rate titrated as per institutional DKA protocol). Alpelisib was temporarily discontinued. A comprehensive workup did not reveal any other etiology or causes for DKA, leading to the diagnosis of alpelisib-induced DKA. Her blood glucose normalized within a day of hospitalization. As the patient's acidemia and hyperglycemia improved, she was discharged on oral antidiabetic medications with close outpatient follow-up of blood glucose with the consultation of an endocrinologist. Figure 2 shows the blood glucose trend of the patient during the current hospitalization.

## Outcome and Follow-up

Post-discharge, the patient was transitioned from insulin to oral antidiabetic medications. Given her poor tolerance to metformin and empagliflozin, she was started on sitagliptin with ideal blood glucose levels. The oncology team attempted to resume alpelisib at 150 mg daily but it was complicated by recurrent hyperglycemia despite a maximal dose of sitagliptin. Thus, alpelisib was permanently discontinued from her oncological treatment regimen in February 2022. Her blood glucose levels remained stable thereafter.

## Discussion

Alpelisib is a selective P13Kα inhibitor and has shown sensitivity to PIK3CA-mutated tumors in patients with advanced solid tumors (5). The efficacy of alpelisib was studied in the SOLAR-1 trial, a phase III, triple-blinded prospective study. The study revealed a statistically significant (*P*-value .00065) progression-free survival in the alpelisib plus fulvestrant cohort (11 months) when compared to the placebo plus fulvestrant group (5.7 months). In addition, a delayed time to first chemotherapy by 8.5 months was noted with the addition of alpelisib to fulvestrant compared to fulvestrant alone (6).

The most frequent adverse event of any grade in the SOLAR-1 trial was hyperglycemia (in 63.7% in the alpelisib-fulvestrant group and 9.8% in the placebo-fulvestrant group). Other common adverse effects were diarrhea, nausea, decreased appetite, rash, vomiting, weight loss, stomatitis, and fatigue. Hyperglycemia was the most common adverse event of grade 3 (fasting plasma glucose [FPG > 250–500 mg/dL (13.88–27.75 mmol/L)] or 4 [FPG >500 mg/dL (27.75 mmol/L)], which occurred in 36.6% of the patients who received alpelisib-fulvestrant and 0.7% of those who received placebo-fulvestrant (3, 6). The most common adverse reaction that led to discontinuation and a dose reduction of alpelisib was hyperglycemia (in 6% and 29% of patients, respectively) (7). The median time to onset of grade 3 hyperglycemia was 15 days (range, 5-395 days) (2). Severe hyperglycemia, with some cases of hyperglycemic hyperosmolar non-ketotic syndrome or ketoacidosis, has been reported in patients with alpelisib treatment (8). Ketoacidosis was a rare adverse effect of alpelisib reported in 0.7% of patients (n = 2) (7). Patients with well-controlled diabetes were eligible to enroll in the SOLAR-1 trial. However, it does not establish the safety of alpelisib in patients with type I and uncontrolled type II diabetes, as these patients were excluded from the trial (8). It was found that individuals who were diabetic or prediabetic at baseline had a more pronounced increase in FPG when compared to those with normal blood glucose levels (2).

The insulin-mediated PI3K activates protein kinase-B (AKT). Activated AKT regulates glucose levels by promoting the translocation of glucose transporter 4 receptors to the cell surface and enhancing glycogenesis in insulin-responsive tissues (9). Alpelisib inhibits the PI3K/AKT pathway and causes hyperglycemia, an on-target effect of alpelisib (3).

Among patients who developed hyperglycemia in the SOLAR-1 trial, 87.1% of patients were managed with metformin alone or metformin in combination with other antihyperglycemic medication (insulin, sulfonylureas, and dipeptidyl peptidase-4 inhibitors). This was associated with a rapid glycemic control with a median of 6 days for improvement by grade ≥1 (range 4-7 days) (2, 8). In addition, among all patients with elevated FPG who continued fulvestrant after discontinuing alpelisib, 96% of patients had their FPG levels returned to baseline (8).

The management of alpelisib-induced hyperglycemia follows specific guidelines discussed in Table 2.

**Table 2.** Guidelines for the management of alpelisib-induced hyperglycemia

Grade of hyperglycemia	Recommended management
Patients with grade 1 and 2 hyperglycemias	Do not require dose adjustments of alpelisib and may require initiating or intensifying antidiabetic medication (metformin). Reduce alpelisib dose by 1 level if FPG does not reduce to grade ≤1 within 21 days.
Patients with grade 3 hyperglycemia [FPG > 250–500 mg/dL (13.88–27.75 mmol/L)]	Managed similarly with the added consideration of an additional drug for 1 to 2 days until hyperglycemia improves and temporary discontinuation of alpelisib, along with the correction of electrolyte abnormalities and intravenous hydration as required. While off alpelisib, if FPG resolves to grade ≤1 within 3 to 5 days, the drug can be resumed with 1 level reduced dose. Alpelisib should be permanently discontinued if the FPG does not resolve to grade ≤1 within 21 days of antidiabetic treatment.
Patients with grade 4 hyperglycemia [FPG > 500 mg/dL (27.75 mmol/L)]	Managed similarly to patients with grade 3. If grade 4 hyperglycemia persists, alpelisib should be permanently discontinued.^2,4^

Abbreviations: FPG, fasting plasma glucose.

Insulin sensitizers (metformin) may be preferred to insulin secretagogues (meglitinide, sulfonylurea) to manage alpelisib-induced hyperglycemia because of insulin spikes and relative resistance observed with P13K inhibitors. Metformin is the first line of treatment and should be started at a dose of 500 mg/day and then increased gradually. Some consider sodium-glucose cotransporter 2 inhibitors as the second-line agent, especially if metformin is not well tolerated due to gastrointestinal adverse effects. However, there is a potential risk of euglycemic DKA. Short-term insulin should be used to manage acute cases and more severe hyperglycemia due to alpelisib and when hyperglycemia is not well controlled with oral anti-hyperglycemia agents alone (2, 4). As alpelisib is a CYP2C9 inducer, glimepiride, glipizide, and glyburide may not effectively manage hyperglycemia (10).

## Learning Points

- Before starting alpelisib treatment, FPG and HbA1c should be tested, and blood glucose should be optimized.
- Individuals who are diabetic or prediabetic at baseline are at a higher risk of developing a more pronounced increase in FPG with alpelisib.
- Once alpelisib treatment is initiated, FPG and HbA1c should be monitored regularly.
- Patients should be advised regarding the signs and symptoms of hyperglycemia, including polyuria, polydipsia, and increased appetite with weight loss.
- Identifying patients potentially at risk for DKA while on alpelisib and standardizing management strategies in patients with limited chemotherapeutic options is significant.

## Data Availability

Original data generated and analyzed during this study are included in this published article.
